# Reconstructive Procedures of the Auricular Concha after Cutaneous Oncologic Surgery: A Systematic Review

**DOI:** 10.3390/jcm12206521

**Published:** 2023-10-14

**Authors:** Sofia Moreno-Vazquez, Javier Antoñanzas, Inés Oteiza-Rius, Pedro Redondo, Rafael Salido-Vallejo

**Affiliations:** 1 School of Medicine, University of Navarra, 31008 Pamplona, Spain; smoreno.8@alumni.unav.es; 2 Department of Dermatology, University Clinic of Navarra, School of Medicine, University of Navarra, 31008 Pamplona, Spain; jantonanzas@unav.es (J.A.); ioteiza@unav.es (I.O.-R.); predondo@unav.es (P.R.)

**Keywords:** systematic review, auricular concha, conchal bowl, cutaneous oncologic surgery, dermatologic surgery, malignant skin neoplasm, reconstructive procedures, complications, aesthetic

## Abstract

Reconstruction of the auricular concha poses a challenge due to its difficult access and limited tissue flexibility; however, there are no recommendations in the literature on which reconstructive technique should be favored for this anatomical site. This systematic review intends to describe and compare the reconstructive techniques used in conchal bowl reconstruction following cutaneous oncologic surgery of this region, with regard to their complications and aesthetic results. In doing so, we aim to identify the best suited reconstructive procedure(s) for the conchal bowl. The six databases searched (PubMed, Scopus, Web of Science, Ovid, SciELO, and CENTRAL) yielded twelve eligible studies that explored the revolving door flap, split-thickness skin grafts (STSG), full-thickness skin grafts (FTSG), second intention healing, the preauricular translocation flap, subcutaneous pedicle grafts, and other local flaps. Qualitative synthesis of the results concluded that the revolving door flap could be the reconstructive procedure of choice for the auricular concha, following skin cancer excision. It has a low risk of necrosis, infection, and postoperative hemorrhage, as well as excellent aesthetic outcomes. STSG may be used as an alternative. Nonetheless, due to the low sample size and the high risk of bias in some studies, further investigations must be conducted on this subject.

## 1. Introduction

There is currently a wide variety of possible reconstructive surgical procedures to be conducted on conchal bowl defects after skin cancer surgery; however, to the knowledge of the authors of this paper, there are no published systematic reviews on this topic, and therefore, no conclusive evidence establishing a specific technique as the gold standard, in terms of complications and aesthetic results. The need for a clear recommendation on which reconstructive technique to implement in the conchal bowl stems from the anatomical challenges of this site, namely, its difficult access and limited tissue flexibility.

**Figure 1:**
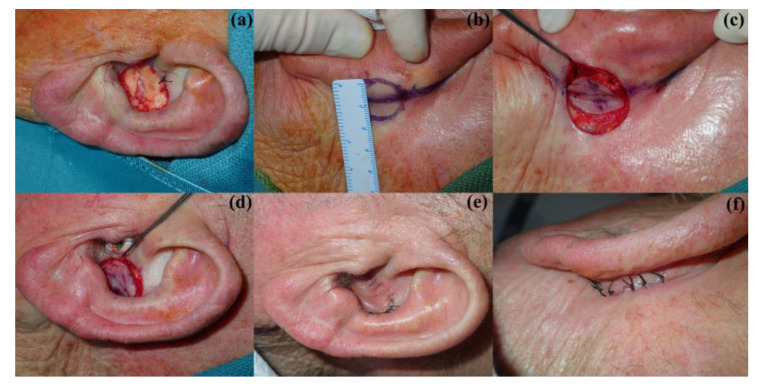
Images depicting the revolving door flap: (**a**) first, the tumor is removed. (**b**) Then, the postauricular flap is designed. (**c**) The flap is carved and a transcartilage incision is made. (**d**) The flap is passed from the postauricular region to the conchal bowl via the transcartilage incision. (**e**) The flap is sutured to the conchal bowl. (**f**) The postauricular incision is sutured.

**Figure 2:**
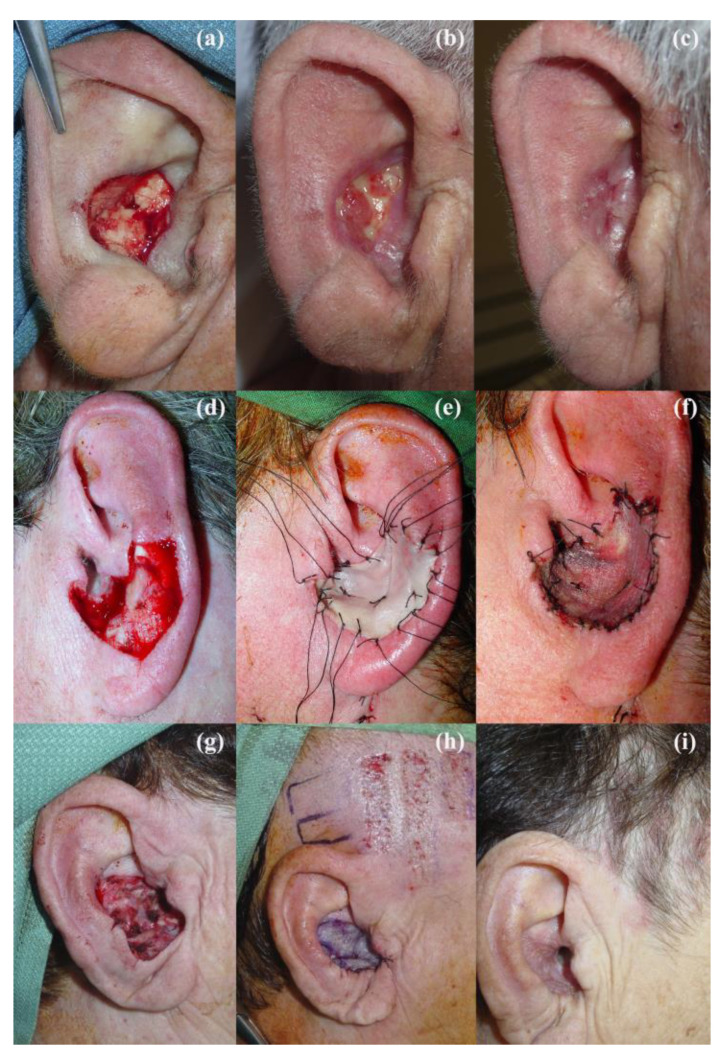
Images depicting several reconstruction techniques used in the conchal bowl area. The top row shows a case of secondary intention healing: (**a**) the defect immediately after tumor excision, (**b**) two weeks later, and (**c**) four weeks later. The middle row shows a case of FTSG: (**d**) the defect immediately after tumor excision, (**e**) with the FTSG sutured onto the defect, and (**f**) five days later. Finally, the bottom row shows a case of STSG taken from the ipsilateral temporal region: (**g**) the defect immediately after tumor excision, (**h**) with the STSG placed over the defect, and (**i**) 4 weeks later, after suture removal. Note how the regrowth of hair follicles in the donor region helps to hide the scar here.

The most used reconstructive procedures described in the literature are the following: the revolving door flap, the split-thickness skin graft (STSG), the full-thickness skin graft (FTSG), second intention healing, and the preauricular translocation flap, as well as other local flaps [1,2]. The revolving door island flap consists of a postauricular flap that is moved to the anterior aspect of the auricle through a transcartilage incision, as depicted in Figure 1 [1,3,4,5,6,7,8,9]. It receives its irrigation from a central vascular pedicle at the postauricular sulcus originating in branches of the posterior auricular artery [3,4,5,6,7,8,9]. STSG, on the other hand, involves taking a graft from a donor site such as the pre- or retroauricular region, scalp, or supraclavicular area, among others, favoring the first two, due to better healing, less infection, and patient preference [10]. FTSG, on the contrary to STSG, involves harvesting a graft containing all the layers of the skin in their entirety, not only the epidermis and superficial dermis [11]. The same donor sites may be used [3]. Dessy (2010) favored the contralateral postauricular region, followed by the supraclavicular and inner arm regions, for FTSG [3]. Secondary intention healing, allowing the defect to close on its own, has also classically been used to reconstruct the auricular concha [12]. Because the wound is initially left with no tissue coverage, appropriate wound care is needed to allow for proper healing [12]. Examples of secondary intention healing, FTSG, and STSG can be seen in Figure 2. Finally, local preauricular flaps may be performed to provide coverage of a conchal bowl defect; the preauricular translocation flap has been commonly used. This flap is raised from the skin anterior to the tragus, and then, rotated over the tragus until it rests over the intertragic notch (previously de-epidermized) and the conchal bowl defect, leaving a pedicle located inferiorly [9]. Because the tissue of the auricular concha and its surrounding areas is lacking in elasticity, local flaps may only be used for small defects [9]. This is the same reason why the direct closure of conchal bowl defects is usually not possible [9].

Possible complications arising from any of the aforementioned reconstructive procedures include flap necrosis, postoperative hemorrhage, infection, wound dehiscence, and altered scarring. The latter includes scar contraction, hypertrophic or keloid scarring, pinning of the ear to the scalp, prominence of the earlobe, dimpling at the postauricular sulcus, and finally, pincushioning or a trap-door effect in which the flap is elevated centrally in comparison to the surrounding skin [13]. While secondary intention healing is often favored due to its simplicity and mostly satisfactory aesthetic results, in larger conchal bowl defects, there could be an increased risk of infection and altered scarring. On the other hand, because grafts and flaps allow for a shorter healing time, they do not have such tendencies to become infected. Still, grafts may result in altered scarring due to central constriction of the graft. Moreover, while local flaps may provide slightly better aesthetic outcomes in this sense, STSG and secondary intention healing allow for better surveillance of any possible cancer recurrences [1]. There is much controversy on the topic, with some authors claiming secondary intention healing does not in fact cause more pain, infection, and poor cosmesis than the alternative reconstructive techniques [12]. Others, however, assert that only the revolving door flap provides adequate coverage when the conchal bowl’s cartilage is left exposed [5]. With no consensus on which reconstructive procedures provide the best outcomes, both regarding complications and aesthetic results, a review of this subject seems extremely relevant.

## 2. Materials and Methods

### 2.1. Data Sources

The databases searched were PubMed, Scopus, Web of Science, Ovid, SciELO, and Central Register of Controlled Trials (CENTRAL). The references of relevant studies identified via the search strategy were also studied by the reviewers as potentially relevant articles. The search was conducted in all databases and reference lists on 10 October 2022.

### 2.2. Search Strategy

The search terms used were the following: ((“Reconstruction”) OR (“reconstructing”) OR (“reconstructive”) OR (“secondary intention”) OR (“second intention”) OR (“split-thickness”) OR (“full-thickness”) OR (flap)) AND ((“auricular skin defects”) OR (“auricular defects”) OR (“auricular conchal”) OR (“postoperative wound infection rates”) OR (“ear defects”) OR (“conchal bowl”) OR (“anterior conchal”)) NOT ((nose) OR (nasal) OR (congenital) OR (orbital) OR (malformation) OR (trauma) OR (microtia)). A publication date filter was used, to include only studies published between 1 January 1995 and 10 October 2022. In two databases, study design filters were also used, in accordance with the exclusion and inclusion criteria. In Scopus, the following were excluded via their corresponding filters: letters, book chapters, notes, conference papers, errata. In Web of Science, books were filtered out.

### 2.3. Inclusion Criteria

The studies included were prospective and retrospective observational clinical studies, case series of more than 5 patients, and randomized and non-randomized control trials published between 1995 and 2022, inclusive. Only articles in English or Spanish were considered. The patient inclusion criteria were the following: patients who had undergone a cutaneous oncologic surgery of the auricular concha and a subsequent reconstructive procedure in the affected area.

### 2.4. Exclusion Criteria

The studies excluded were reviews, systematic reviews, meta-analyses, book chapters, case series of 5 or fewer patients, duplicate or overlapping studies, guidelines, abstracts, lectures, and animal studies. Studies regarding the reconstruction of congenital ear malformations, traumatic ear defects, or any defects other than of the oncologic variety were likewise excluded. Articles on the topic of inner ear surgeries were also disregarded. Finally, studies not regarding the conchal bowl area, or not providing any data on complications or aesthetic results, were excluded, as well.

### 2.5. Study Selection

Studies were selected for inclusion by two independent reviewers, using COVIDENCE^®^. All duplicates within the studies yielded by the search strategy were automatically removed by COVIDENCE^®^. The remaining articles were screened by the two reviewers by title and abstract. Subsequently, the full text of the selected studies was accessed, and the two reviewers assessed the reports for eligibility, in accordance with the established eligibility criteria. Disagreements between the reviewers were resolved via dialogue until a consensus was reached, with no need for consulting a third independent reviewer to settle any discrepancies.

### 2.6. Data Extraction

The reports deemed eligible for inclusion were read by two independent reviewers for data extraction (SMV and RSV). The data items obtained from each study included study identification (author, year of publication, and study design), study population (country, sample size, mean age, female percentage, skin neoplasm percentages, surgical defect size, and mean follow-up time), and reconstructive procedure(s) used. Furthermore, outcome data were extracted, specifically, the absolute number of complications or complication incidences (depending on the study) for each reconstructive procedure, and aesthetic results (on a numerical scale) from patient and physician perspectives for each reconstructive procedure. The complications considered were postoperative hemorrhage, altered scarring, infection, necrosis, and dehiscence. As it was not the purpose of this article, recurrence rates were not considered as an outcome and consequently not included in the database.

### 2.7. Risk-of-Bias Assessment

Two independent authors used the following tools to assess risk of bias in the included studies: RoB 2.0 for randomized control trials, JBI Critical Appraisal Checklist for Case Series, JBI Critical Appraisal Checklist for Quasi-experimental Studies, and Newcastle–Ottawa Scale for case–control and cohort studies [14,15,16,17].

### 2.8. Data Synthesis

The mean incidence of each complication type for each reconstructive technique was calculated for studies that only provided absolute numbers of complications, namely, Wines (2001) [2], Futoryan (1995) [18], Levin (1996) [12], Thuile (2018) [10], Zhu (2016) [9], Talmi (1996) [8], Dyson (2019) [4], and Golash (2020) [5]. Iljin (2016) [6] and Iljin (2018) [7] directly reported this effect measure [6,7]. Regarding aesthetic results, for reports presenting this outcome as a score on a numerical scale (Franco-Muñoz (2020) [1], Dessy (2010) [3], and Thuile (2018) [10]), the mean of these proportions for all patients and for all surgeons was taken. Due to a lack of homogeneity regarding outcome data among the included studies, a meta-analysis was not conducted. Instead, a qualitative analysis of the data extracted was performed.

## 3. Results

### 3.1. Study Characteristics

**Figure 3:**
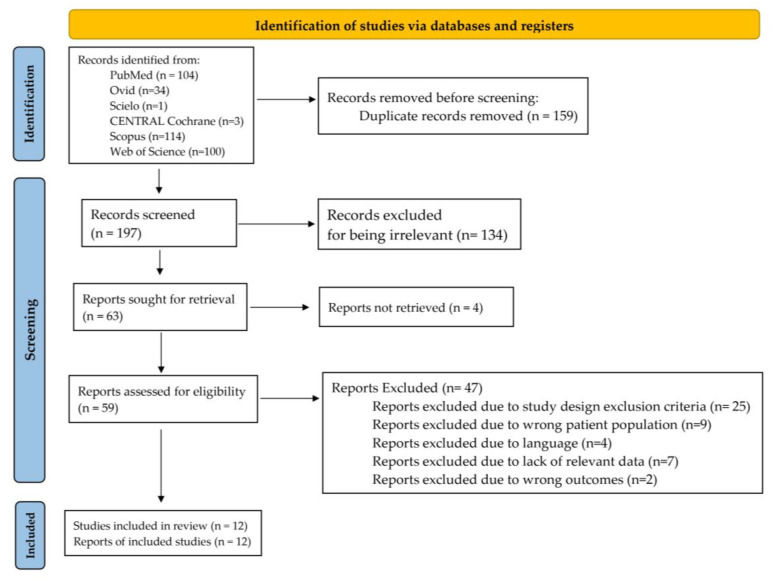
Flow diagram depicting the study selection process.

The initial search strategy yielded a total of 197 references, not including duplicate records. Ultimately, twelve studies were deemed eligible for enrollment, following the selection process depicted in Figure 3. All data extracted from the enrolled studies, including study characteristics and outcomes, are presented in Table 1 and Table 2. Not all included studies presented data on the two outcomes considered in this review [1,2,3,4,5,6,7,8,9,10,12,18]. Moreover, while complications were presented in the form of incidences in all included studies, aesthetic results were reported in a heterogeneous manner [1,2,3,4,5,6,7,8,9,10,12,18]. For instance, among the five studies examining this outcome after reconstruction with a revolving door flap, Iljin (2018) [7], Iljin (2016) [6], Franco-Muñoz (2020) [1], and Dessy (2010) [3] used satisfaction scales to record the aesthetic outcomes, whereas Zhu (2016) [9] only provided a qualitative description [1,3,6,7,9]. Furthermore, the satisfaction scales used in each study differed [1,3,6,7].

### 3.2. Risk of Bias

The results of the risk-of-bias assessments for all enrolled studies can be seen summarized in Table 3. Overall, the risk of bias for the enrolled case series was low following assessment with the JBI Critical Appraisal Checklist for Case Series [1,4,5,6,7,8,9,10,12,16]. However, Talmi (1996) [8] scored 6/10, rendering its risk of bias concerning [8,16]. Its main issue was a lack of clear inclusion criteria for the study’s participants, as well as its failure to have consecutive and complete inclusion [8,16]. On the other hand, Thuile (2018) exhibited high methodological quality, as it scored a 9/10 in the risk-of-bias assessment [11,17]. In stark opposition, Futoryan (1995) [18] and Wines (2001) [2], the cohort studies, performed poorly in the risk-of-bias assessment, scoring 4/9 in the Newcastle–Ottawa Scale, [2,14,18]. While they scored very well in the “selection” category, this was not enough to outweigh the lack of data on follow-up times and losses, and the absence of any attempts to control for potential confounding factors [2,14,18]. Finally, the assessment of the only RCT, Dessy (2010) [3], with Cochrane’s RoB 2.0 tool raised some concerns regarding risk of bias, although it did not reach a high level of risk of bias [17]. The study failed to specify whether allocation sequence concealment had been kept, and it did not discuss the baseline characteristics of the two intervention groups; therefore, there was no evidence to confirm whether the random allocation process had been successful in controlling for confounding factors [3,17]. In addition, it was unclear whether the analysis plan yielding the study’s results was fully established prior to the unblinded results becoming available [3,17]. All other aspects of the randomized control trial had low risk of bias. Since there were no missing data, the outcome (aesthetic results) was measured adequately, and there were no deviations from the assigned interventions [3,17].

### 3.3. Revolving Door Flap

Talmi et al. reported only one case of infection after performing 11 revolving door flaps (9.1%), whereas other more recent studies (Zhu (2016), Iljin (2018), Iljin (2016), Dyson (2019)) documenting this complication did not find any cases of infection [4,6,7,8,9].

Regarding altered scarring, in Iljin (2016), pinning of the ear was identified in 15.4% of cases, but there was no incidence of flap constriction, flap depression, or auditory canal stenosis [6]. On the other hand, a more recent case series, Iljin (2018), reported 21.1% of cases with pinning of the auricle, 15.8% of cases with prominent earlobes, and 15.8% of cases with auditory canal narrowing [7]. In Dyson (2019), pincushioning occurred in 6.4% of cases, although no dimpling at the pedicle site or pinning of the auricle developed [4]. Finally, in the most recent study, Golash (2020), the only form of altered scarring resulting from the reconstruction was pinning of the ear [5]. All seven patients in this case series developed this complication; however, the pinning was only considered major in three cases [5]. In juxtaposition, Dessy (2010) did not find any cases of altered scarring [3].

Regarding postoperative hemorrhage, only one study reported on this complication—Dyson (2019)—and it found no cases of such an event [4]. Likewise, necrosis or failure of the flap or graft did not occur in any of the six papers that contemplated this complication [3,4,5,6,8,9]. Dehiscence was only considered as a potential complication in Talmi (1996), where 18.2% of cases resulted in this outcome; however, it must be noted that these patients were well above the mean age [8].

The method for reporting aesthetic results differed in each of the five studies examining this outcome [1,3,6,7,9]. In Franco-Muñoz (2020), average patient satisfaction was 4.71 out of 5, and average dermatologist satisfaction was 4.29 out of 5 [1]. The aesthetic results in Dessy (2010) were similar: average patient satisfaction was 9.40 out of 10, while average physician overall satisfaction was 9.53 out of 10, and average physician color–texture match evaluation yielded a score of 8.98 out of 10 [3]. In both Iljin (2018) and Iljin (2016), there was complete patient–surgeon consensus [6,7]. In the earlier publication, there was complete satisfaction in 84.6% of cases, and moderate satisfaction in 15.4% of cases, while in the later publication, there was complete satisfaction in 57.9% of cases and moderate satisfaction in 42.1% of cases [6,7]. The aesthetic results in Zhu (2016) were described as “good”, and all patients were said to be satisfied; however, the surgeon’s opinion was not recorded in this study [9].

### 3.4. Secondary Intention Healing

Three of the included studies examined the complications arising from this technique [2,12,18]. Futoryan (1995) [18] reported no cases of infection, while 7.1% of patients in Levin (1996) and 2.8% of patients in Wines (2001) developed a postoperative infection [2,12]. In addition, Levin (1996) reported altered scarring in the form of webbing in 14.2% of cases [12]. Furthermore, Wines (2001) documented postoperative hemorrhage in 2.8% of cases, but no cases of necrosis or altered scarring [2]. The aesthetic outcomes of secondary intention healing were only recorded in Levin (1996) [12]. According to the surgeons, unacceptable healing with web formation occurred in 14.3% of patients, while an excellent or acceptable aesthetic result was achieved in the remaining 85.7% of patients [12].

### 3.5. Split-Thickness Skin Graft

Three studies reported on the complications occurring after reconstruction with STSG [2,10,18]. Wines (2001) noted graft necrosis in 3.9% of patients, infection in 1.3% of patients, altered scarring in 1.4% of patients (half due to pincushioning, and half due to graft contraction), and postoperative hemorrhage in 1.3% of patients [2]. Similarly, Thuile (2018) documented some cases of infection and postoperative hemorrhage, although the incidence of these was higher than in Wines (2001): 22.2% of patients developed an infection (11.1% occurring at the donor site, and the other 11.1% at the receptor site), and 11.1% of patients suffered from postoperative hemorrhage [2,10]. On the contrary to the findings of Wines (2001), Thuile (2018) did not identify any cases of altered scarring, and Futoryan (1996) did not find any cases of infection [2,10,18]. Only Thuile (2018) examined the aesthetic outcomes of STSG [10]. The average patient satisfaction and the aesthetic outcome according to one dermatologist and two plastic surgeons was rated as 9.78 out of 10, 9.22 out of 10, and 9.11 out of 10, respectively [10].

### 3.6. Full-Thickness Skin Graft

Three of the included studies examined the complications resulting from FTSG reconstruction: Wines (2001) found that only 3.9% of patients presented graft failure, with no cases of infection, altered scarring or postoperative hemorrhage [2]. On the other hand, Futoryan (1995) reported a steep 33.3% incidence of infection [18]. Nonetheless, this percentage corresponded to only two cases of infection [18]. Out of the six patients whose auricular conchas were reconstructed using an FTSG in Futoryan (1995), only two received antibiotic prophylaxis; therefore, among four patients without antibiotic prophylaxis, half developed an infection [18]. These patients did not present larger or deeper preoperative conchal bowl defects [18]. Unfortunately, patient comorbidity data, which might have justified the development of an infection, were unavailable [18]. Dessy (2010) recorded a high incidence of partial graft necrosis (30%), whereas no cases of total graft failure were documented [3]. Unlike Wines (2001) [2], Dessy (2010) [3] did find a high incidence of postoperative hemorrhage (20% of patients), and of altered scarring in the form of external auditory canal stenosis (20% of patients) [3]. The aesthetic outcomes of FTSG were only documented in Dessy (2010) [3]. Average patient satisfaction was rated 7.00 out of 10, while average physician overall satisfaction was 6.80 out of 10, and average physician color–texture match evaluation yielded a score of 6.87 out of 10 [3].

### 3.7. Other Reconstructive Procedures

Wines (2001) studied various other methods of reconstruction which were not examined in any of the other included studies: side-to-side repair, subcutaneous pedicle graft, rotation flap, and advancement flap [2]. Their findings showed no cases of infection, or flap necrosis, with any of the aforementioned flaps and grafts, but a 4.0% incidence of pincushioning and of postoperative hemorrhage both with the subcutaneous pedicle graft [2]. Lastly, the preauricular transposition flap was studied both by Wines (2001) and Zhu (2016) [3,10]. While the former found a 16.7% incidence of flap failure, the latter did not identify any cases of such an outcome [2,9]. Furthermore, both studies reported no cases of infection, and Wines (2001) also found no incidence of altered scarring or postoperative hemorrhage [2,9]. According to Zhu (2016), the preauricular transposition flap also yielded aesthetically satisfactory outcomes [9].

## 4. Discussion

It may be concluded that the revolving door flap has little tendency to develop infections. The only case of infection among the studies reporting on this flap’s complications involved exceptional circumstances: the patient had inadequate perioperative wound care [4,6,7,8,9]. Furthermore, this flap has excellent survival rates, as all six studies recording flap necrosis incidence found no cases of this complication [3,4,5,6,8,9]. Although the incidence of postoperative hemorrhage was also nonexistent, because this complication was only studied in a single paper, these findings are not entirely reliable [4]. Similarly, the 18.2% incidence of surgical wound dehiscence found in Talmi (1996) may not be accurate as it was the only study considering this outcome, and it only examined eleven cases [8]. Considering all possible complications, the revolving door flap has yielded excellent results [3,4,5,6,7,8,9]. Specifically, its advantages include a low-to-null incidence of necrosis and infection; however, its main disadvantage is its tendency to produce pinning of the auricle to the scalp [3,4,5,6,7,8,9]. Despite this limitation, its overall aesthetic results ranged from excellent to moderately satisfactory, the majority gathering at the better end of the spectrum [1,3,6,7,9]. Moreover, the results regarding aesthetic outcome seem reliable, as they were obtained from five different studies, all of considerable sample size (13 to 40 patients), with the exception of Franco-Muñoz (2020), which included only seven patients [1,3,6,7,9].

Unlike the revolving door flap, secondary intention healing seemed to predispose patients to infection of the conchal bowl, as two studies with considerable sample size found cases of infection, albeit at a low incidence [2,12]. Although in Futoryan (1995), no patients developed an infection, this discrepancy might be explained by its significantly smaller sample size [18]. Moreover, it seems reasonable that second intention healing would have a higher risk of infection as the wound remains without coverage for a longer period, in comparison to flaps and grafts [2,12]. As a result, proper wound care becomes fundamental [2,12]. The results regarding altered scarring with secondary intention healing were contradictory, as Levin (1996) found a considerable incidence of webbing, whereas Wines (2001) did not identify any cases of such complication [2,12]. Postoperative hemorrhage incidence was low, necrosis was nonexistent, and aesthetic outcomes were mostly satisfactory; therefore the main issue with secondary intention healing seems to be the risk of infection [2,12,18]. This finding coincides with the literature on this topic, as many authors have voiced their concerns with infection rates when using secondary intention healing [1]. Nonetheless, the data in this review on postoperative hemorrhage and cosmesis with secondary intention healing is limited, as only one study reported on each of these; therefore, further investigations are required on this subject [2,12]. Still, in practice, the cosmetic results obtained with secondary intention healing are satisfactory and it may be considered an adequate reconstructive option in select patients, especially those with high surgical morbidity, or in high-risk tumors to allow for easier detection of local recurrences [19].

Among the various reconstructive methods examined by the included studies, STSG was one of two techniques to present cases of all complications [2,10,18]. Admittedly, their incidence was low in Wines (2001); however, postoperative hemorrhage and infection incidence was considerable in Thuile (2018) [2,10]. Despite these complications, aesthetic outcomes were excellent, both from patient and surgeon perspectives [10]. Nevertheless, cosmetic results were extracted from a single study with a reduced sample size, and as such, could be skewed [10]. Thus, more RCTs and cohort studies on this matter should be conducted.

Similarly to STSG, FTSG caused a wide variety of complications; however these findings were discordant across studies [2,3,18]. While Wines (2001) found no cases of infection, altered scarring, or postoperative hemorrhage, the other two papers studying this technique found a high incidence of these events [2,3,18]. The discrepancy regarding postoperative hemorrhage could be justified by the administration of subcutaneous heparin in the majority of patients who developed this complication in Dessy (2010) [2,3]. Nonetheless, controversy remains with respect to the incidence of infection and altered scarring, therefore demanding more evidence on this subject [2,3,18]. FTSG yielded significantly worse cosmetic results than all other reconstructive methods, albeit still decent, presumably due to its many deriving complications [3]. Although only Dessy (2010) reported on the aesthetic outcomes of FTSG, this study was an RCT with a considerable sample size, and it inquired about both patient and surgeon opinions [3].

More evidence is needed regarding the outcome of local flaps as only Wines (2001) studied these, and the sample size for each of these was very limited [2]. The same limitation is true for the subcutaneous pedicle graft, although the data provided by Wines (2001) establishes it as a valid alternative, as it does not cause necrosis or infection, and only has a low incidence of pincushioning and postoperative hemorrhage [2].

Controversy remains regarding the preauricular transposition flap, as Wines (2001) reported a considerable incidence of flap failure, but Zhu (2016) did not identify any cases of this complication [2,9]. Therefore, more studies examining this flap are needed. Otherwise, this technique seems acceptable, as no cases of infection, altered scarring, or postoperative hemorrhage were recorded, and it was aesthetically satisfactory [2,9].

**Figure 4:**
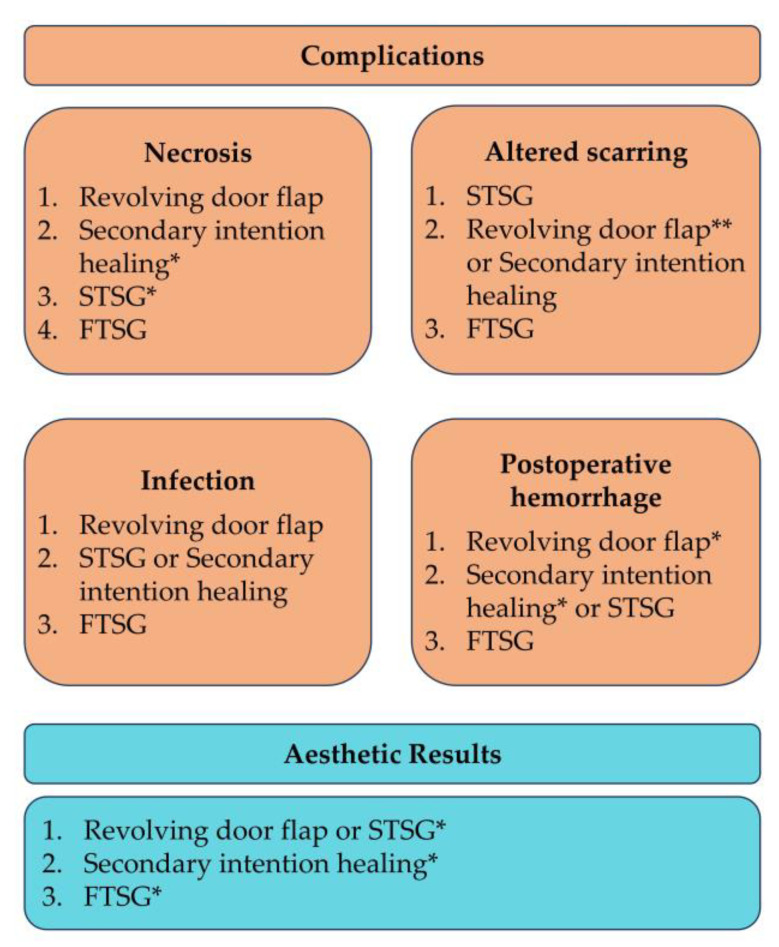
Order of preference of reconstructive procedures based on complication rates and aesthetic results of the included studies, as shown in Table 2. Data on dehiscence were limited to one study, which only examined the revolving door flap; therefore no comparisons can be made regarding this complication. * Only one of the included studies presented data on this. ** Pinning of the ear was the main issue with scarring.

Although all existing reconstructive procedures yield acceptable aesthetic outcomes, the best results were achieved when using the revolving door flap or STSG [1,3,6,7,9,10,12]. However, given that STSG more frequently results in complications, the revolving door flap may be considered the reconstructive procedure of choice for the conchal bowl region, in terms of complications and cosmetic results [2,3,4,5,6,7,8,9,10,18]. STSG may be considered a suitable alternative to the revolving door flap [2,3,4,5,6,7,8,9,10,18]. Other techniques, such as secondary intention healing and FTSG, may be used but taking into account some relevant aspects [2,3,12,18]. Secondary intention healing poses a greater risk of infection, as compared to the revolving door flap [2,3,4,5,6,7,8,9,12,18]. Likewise, its aesthetic outcomes are inferior to those of the revolving door flap [1,3,6,7,9,12]. FTSG seems to have a tendency to cause complications of all kinds, a propensity that negatively impacts its aesthetic outcomes [2,3,18]. Although STSG also has a proclivity to cause complications, it should be favored over FTSG due to its comparatively lower incidence of complications and superior cosmetic results [2,3,10,18]. Figure 4 summarizes our review’s findings.

However, there are several relevant considerations to discuss regarding the reconstruction of oncological defects in the auricular concha. This systematic review has exclusively focused on assessing aesthetic outcomes and complication rates, but in routine surgical practice, other circumstances must be taken into account. The margin status is a major concern in all skin cancer patients, and its clearance should be guaranteed before reconstruction. If there is clinical or radiological suspicion of tumor involvement of the cartilage, then it may need to be excised, which influences the reconstruction. In cases of high-risk tumors (aggressive histological subtype, recurrent tumors, proximity to the external auditory canal, among others) where Mohs surgery cannot be performed, the decision on reconstruction should prioritize oncological outcomes. Therefore, deferred closures until clear margins are achieved or the use of grafts instead of local flaps would be recommended to ensure better postoperative monitoring. Another important consideration when selecting a reconstructive method in the auricular concha is the presence of exposed cartilage with or without the perichondrium. In cases where the perichondrium could not be preserved during excision, the viability of graft placement may be compromised. Therefore, it is recommended to perform perforations or complete removal of the underlying cartilage before graft apposition or to consider the use of a local flap.

Certainly, the limitations of this review must be discussed. Although the use of dermal regeneration templates may also serve as a useful reconstructive method in large conchal defects, this option was not considered in this review; however, a later search in PubMed revealed no published articles on the topic. Regarding the individual studies included, most were case series, which do not tend to provide the highest quality of scientific evidence [1,4,5,6,7,8,9,10,12]. Unfortunately, only one RCT and two cohort studies met the eligibility criteria [2,3,18]. In addition, approximately half of the studies included had a considerably low sample size [1,2,3,4,5,6,7,8,9,10,12,18]. Some articles also predated the 2000s, thus potentially providing obsolete data [8,12,18]. The reason for including these studies was that certain well-established techniques, such as secondary intention healing, have rarely been revisited in recent years. To avoid including excessively outdated studies, the eligibility criteria excluded papers published prior to 1995. Furthermore, the manner in which some articles presented aesthetic outcomes was also inadequate: Zhu (2016) and Levin (1996) did not use objective numerical methods of evaluating cosmetic results [9,12]. Consequently, it became difficult to compare aesthetic outcomes across studies. As discussed previously, some studies raised concerns regarding risk of bias, namely, the two cohort studies [1,2,3,4,5,6,7,8,9,10,12,14,15,16,17,18]. Any limitations in the included studies are of course transferred to the review; however, beyond these, other weaknesses must be considered, fundamentally, that a meta-analysis was not conducted, with only a qualitative analysis carried out instead. Because of the manner in which the papers reported their outcomes, the included studies were too heterogeneous, and performing a meta-analysis was not possible [1,2,3,4,5,6,7,8,9,10,12,18]. Finally, it must be noted that this review analyzed reconstructive procedures solely from the perspectives of complications and aesthetic results. However, those patients with high-risk cutaneous tumors may benefit from secondary intention healing or grafts, instead of flaps, to more easily detect possible local recurrences.

Despite its weaknesses, this review has various strengths, as well. For instance, most included studies had a long follow-up period, ensuring that even long-term complications and aesthetic defects were detected [1,2,3,4,5,6,7,8,9,10,12,18]. Moreover, this review can provide a global perspective as it comprises studies from several countries conducted by different specialists (dermatologists, otorhinolaryngologists, or plastic surgeons) [1,2,3,4,5,6,7,8,9,10,12,18]. More importantly, all existing reconstructive procedures applicable to the auricular concha were explored [1,2,3,4,5,6,7,8,9,10,12,18]. Equally important, the PRISMA guidelines were closely followed when completing this review [20].

The results of our systematic review suggest that the revolving door flap could be the most suited reconstructive procedure for the auricular concha according to its low risk of complications and its excellent aesthetic outcomes. Second intention healing, STSG, and FTSG have a worse safety profile, but they still represent a valuable reconstructive option in determinate cases of high-risk tumors and may be used as an alternative. Nonetheless, more controlled clinical trials are needed to increase the low amount of clinical evidence currently available.

## Figures and Tables

**Table 1:** Characteristics of included studies.

Author (Year)	Study Design	Country	Sample Size	Mean Age (Years)	Mean Follow-Up (Months)	Female %	Skin Neoplasm(s)	Defect Size after Surgical Excision (Largest Diameter in cm) *	Reconstructive Procedure(s)
Wines (2001) [2]	Retrospective cohort	Australia	272	60.0	Not reported	8.1	76% BCC 10% SSC 4% Bowen’s Disease	SI: <1.0–>6.0 STSG: <1.0–5.9 FTSG: <1.0–2.9 SCP: <1.0–2.9 Side-to-side repair: <1.0–1.9 TP: <1.0–1.9 RP: 1.0–1.9 AF: <1.0–>6.0	Secondary intention healing (SI) Split-thickness skin graft (STSG) Full-thickness skin graft (FTSG) Subcutaneous pedicle graft (SCP) Side-to-side repair Transposition flap (TP) Rotation flap (RP) Advancement flap (AF)
Futoryan (1995) [18]	Retrospective cohort	USA	8	Not reported	Not reported	Not reported	12.5% SCC 82.5 % BCC	STSG: 3.6 FTSG: 2.8 SI: 1.4	STSG FTSG SI
Franco-Muñoz (2020) [1]	Case Series	Spain	7	80.0	40	14.3	28.6% SCC 71.4% BCC	1.3	Revolving Door Flap
Dessy (2010) [3]	Randomized Control Trial	Italy	40	64.7	24	30.0	75% BCC 22.5% SCC 2.5% Melanoma	2.0–4.0	Revolving Door Flap vs. FTSG
Levin (1996) [12]	Case Series	USA	14	Not reported	6	Not reported	Not reported	3.1 cm^2^ **	SI
Thuile (2018) [10]	Case Series	Italy	9	72.2	34.5	22.2	77.8% BCC 11.1% SCC 11.1% Bowen’s Disease	3.1	STSG
Iljin (2018) [7]	Case Series	Poland	19	71.0	24	31.6	63.2% BCC 36.8% SCC	2.0–5.0	Revolving Door Flap
Iljin (2016) [6]	Case Series	Poland	13	63.0	24	23.0	61.5% BCC 38.5% SCC	2.0–5.0	Revolving Door Flap
Zhu (2016) [9]	Case Series	China	16	Not reported	35	Not reported	81% BCC 19% SCC	PTF: - Revolving Door Flap: 1.5–3.0	Preauricular translocation flap (PTF) vs. Revolving Door Flap
Talmi (1996) [8]	Case Series	Israel	11	70.5	Not reported	18.0	73% BCC 18% SCC 9% Melanoma	1.5–6.0	Revolving Door Flap
Dyson (2019) [4]	Case Series	U.S.A.	94	Not reported	Not reported	Not reported	Not reported	Not reported	Revolving Door Flap
Golash (2020) [5]	Case Series	India	7	58.4	12	43.0	100% BCC	3.0	Revolving Door Flap

* Where ranges are presented the corresponding study did not provide specific defect size data. Where a number is provided this represents the average largest diameter of defects. ** Only data on area of defect size was provided by this study.

**Table 2:** Outcome data extracted from included studies.

Author (Year)	Complications (Incidence among Patients)	Aesthetic Results
Wines (2001) [2]	Flap/Graft Failure: 6/152 (3.9%) of STSG 3/32 (9.4%) of FTSG 2/12 (16.7%) of TP Infection: 2/152 (1.3%) of STSG 1/36 (2.8%) of SI Altered scarring: Pincushioning: 1/152 (0.7%) of STSG 1/25 (4.0%) of SCP Contraction: 1/152 (0.7%) of STSG Postoperative hemorrhage: 2/152 (1.3%) STSG 1/36 (2.8%) of SI 1/25 (4.0%) of SCP	Not reported
Futoryan (1995) [18]	Infection: 2/6 (33.3%) of FTSG	Not reported
Franco-Muñoz (2020) [1]	Not reported	Scale of satisfaction (out of 5): 4.71/5 patient satisfaction 4.29/5 dermatologist satisfaction
Dessy (2010) [3]	Revolving Door Flap Total flap necrosis: 0/20 Partial flap necrosis: 0/20 Altered scarring: Depression in contour of flap: 0/20 External auditory canal stenosis: 0/20 FTSG Total graft necrosis: 0/20 Partial graft necrosis: 6/20 (30%) Altered scarring: External auditory canal stenosis: 4/20 (20%) Depression in contour of flap: 0/20 Postoperative hemorrhage: 4/20 (20%)	* VAS Scale (out of 10): With Revolving Door Flap Patient satisfaction: 9.40/10 Physician evaluation of overall outcomes: 9.53/10 Physicians’ evaluation of color and texture match: 8.98/10 With FTSG Patient satisfaction: 7.00/10 Physician evaluation of overall outcomes: 6.80/10 Physicians’ evaluation of color and texture match: 6.87/10
Levin (1996) [12]	Infection: 1/14 (7.1%) Altered scarring: 2/14 (14.3%)	Excellent/acceptable/unacceptable (by authors’ consensus): Excellent or acceptable: in 12/14 patients (85.7%) Unacceptable (due to web formation): 2/14 (14.3%)
Thuile (2018) [10]	Complications at < 3 months Infection of donor site: 1/9 (11.1%) Postoperative hemorrhage: 1/9 (11.1%) Complications at >3 months: Infection of receptor site: 1/9 (11.1%) Altered scarring (hypertrophic scar): 0/9	VAS Scale (out of 10): 9.78/10 satisfaction among patients 9.22/10 aesthetic outcome according to 1 dermatologist 9.11/10 aesthetic outcome according to 2 plastic surgeons
Iljin (2018) [7]	Infection: 0/19 Altered scarring: Abnormal wound healing: 0/19 Secondary flap shrinkage: 0/19 Depression in contour of flap: 0/19 Pinning of ear: 4/19 (21.1%) Prominent earlobe: 3/19 (15.8%) Auditory canal constriction: 3/19 (15.8%) Venous congestion: 5/19 (26.3%)	Scale of Satisfaction (completely/very/moderately/slightly/not satisfied): 11/19 (57.9%) completely satisfied among plastic surgeon and patients 8/19 (42.1%) moderately satisfied among plastic surgeon and patients
Iljin (2016) [6]	Flap necrosis: 0/13 Infection: 0/13 Altered scarring: Abnormal wound healing: 0/13 Secondary flap shrinkage: 0/13 Depression in contour of flap: 0/13 Auditory canal constriction: 0/13 Pinning of ear: 2/13 (15.4%) Venous congestion: 2/13 (15.4 %)	Scale of Satisfaction (completely/satisfied/moderately/slightly/not satisfied): 11/13 (84.6%) completely satisfied among plastic surgeon and patients 2/13 (15.4%) moderately satisfied among plastic surgeon and patients
Zhu (2016) [9]	Flap necrosis: 0/16 Infection: 0/16	“Aesthetic outcomes were good” - Unnoticeable donor-site scars - Adequate color and texture match - No auricular deformity
Talmi (1996) [8]	Flap failure: 0/11 Infection: 1/11 (9.1%) Dehiscence: 2/11 (18.2%)	Not reported
Dyson (2019) [4]	Flap necrosis: 0/94 Infection: 0/94 Postoperative bleeding: 0/94 Altered scarring: Dimpling at pedicle site: 0/94 Pinning requiring revision surgery: 0/94 Pincushioning: 6/94 (6.4%)	Not reported
Golash (2020) [5]	Flap necrosis: 0/7 Flap congestion: 3/7 (42.9%) Altered scarring: Minor Pinning of ear: 4/7 (57.1%) Major Pinning of ear: 3/7 (42.9%)	Not reported

* 12 months after surgery.

**Table 3:** Risk of bias assessment of included studies.

Author (Year)	Study Design	Risk of Bias Assessment Tool	Result of Risk of Bias Assessment
Futoryan (1995) [18]	Retrospective cohort	Newcastle-Ottawa Scale for Cohort Studies	4/9 stars
Wines (2001) [2]	Retrospective cohort	Newcastle-Ottawa Scale for Cohort Studies	4/9 stars
Dessy (2010) [3]	Randomized Control Trial	RoB 2.0	Some concerns
Levin (1996) [12]	Case Series	JBI Critical Appraisal Checklist for Case Series	8/10 yes, 1/10 no, 1/10 NA
Talmi (1996) [8]	Case Series	JBI Critical Appraisal Checklist for Case Series	6/10 yes, 3/10 no, 1/10 NA
Iljin (2016) [6]	Case Series	JBI Critical Appraisal Checklist for Case Series	8/10 yes, 1/10 no, 1/10 NA
Zhu (2016) [9]	Case Series	JBI Critical Appraisal Checklist for Case Series	7/10 yes, 2/10 no, 1/10 NA
Iljin (2018) [7]	Case Series	JBI Critical Appraisal Checklist for Case Series	8/10 yes, 1/10 no, 1/10 NA
Thuile (2018) [10]	Case Series	JBI Critical Appraisal Checklist for Case Series	9/10 yes, 1/10 NA
Dyson (2019) [4]	Case Series	JBI Critical Appraisal Checklist for Case Series	8/10 yes, 1/10 no, 1/10 NA
Franco-Muñoz (2020) [1]	Case Series	JBI Critical Appraisal Checklist for Case Series	8/10 yes, 1/10 no, 1/10 NA
Golash (2020) [5]	Case Series	JBI Critical Appraisal Checklist for Case Series	7/10 yes, 2/10 no, 1/10 NA

## Data Availability

No new data were created or analyzed in this study. Data sharing is not applicable to this article.
